# Genomic divergence within non-photosynthetic cyanobacterial endosymbionts in rhopalodiacean diatoms

**DOI:** 10.1038/s41598-017-13578-8

**Published:** 2017-10-12

**Authors:** Takuro Nakayama, Yuji Inagaki

**Affiliations:** 1 Center for Computational Sciences, University of Tsukuba, 1-1-1 Tennoudai, Tsukuba, Ibaraki, 305-8577 Japan; 2 Present Address: Graduate School of Life Sciences, Tohoku University, 6-3 Aoba, Aramaki, Aoba-ku, Sendai, Miyagi, 980-8578 Japan

## Abstract

Organelle acquisitions via endosymbioses with prokaryotes were milestones in the evolution of eukaryotes. Still, quite a few uncertainties have remained for the evolution in the early stage of organellogenesis. In this respect, rhopalodiacean diatoms and their obligate cyanobacterial endosymbionts, called spheroid bodies, are emerging as new models for the study of organellogenesis. The genome for the spheroid body of *Epithemia turgida*, a rhopalodiacean diatom, has unveiled its unique metabolic nature lacking the photosynthetic ability. Nevertheless, the genome sequence of a spheroid body from a single lineage may not be sufficient to depict the evolution of these cyanobacterium-derived intracellular structures as a whole. Here, we report on the complete genome for the spheroid body of *Rhopalodia gibberula*, a lineage distinct from *E. turgida*, of which genome has been fully determined. Overall, features in genome structure and metabolic capacity, including a lack of photosynthetic ability, were highly conserved between the two spheroid bodies. However, our comparative genomic analyses revealed that the genome of the *R. gibberula* spheroid body exhibits a lower non-synonymous substitution rate and a slower progression of pseudogenisation than those of *E. turgida*, suggesting that a certain degree of diversity exists amongst the genomes of obligate endosymbionts in unicellular eukaryotes.

## Introduction

Photosynthesis and aerobic respiration were introduced to eukaryotes through the acquisition of plastids and mitochondria, respectively. The two metabolic abilities were primarily innovated among prokaryotes and incorporated into the eukaryotic cellular system via endosymbioses of a photosynthetic bacterium and a bacterium generating energy under the presence of oxygen. Thus, the evolutionary process that transformed an endosymbiotic bacterium into a host-controlled organelle (organellogenesis) is considered one of the critical phenomena in understanding the origin and diversification of modern eukaryotes. Although much effort has been taken to elucidate how organellogenesis proceeded by studying the extant eukaryotes harbouring mitochondria and/or plastids, many uncertainties remained for the early stages of organellogenesis. As both mitochondria and plastids have already been established as organelles, the systems for maintenance and functions of the two organelles in modern eukaryotes most likely retained little information regarding the early process of organellogenesis. In this respect, unicellular eukaryotic cells hosting bacterial intracellular symbionts have been paid attention as new model systems to study organellogenesis^1,2^. These symbionts were established more recently than mitochondria or plastids in eukaryotic evolution, and have been anticipated to be more informative for studying organellogenesis.

Diatoms belonging to the family Rhopalodiaceae have been nominated as one of the new models for studying organellogenesis^2–4^. The Rhopalodiaceae is a group of pennate diatoms that includes three genera, namely *Rhopalodia*, *Epithemia*, and *Protokeelia* ^5^. The genera *Rhopalodia* and *Epithemia* are known to possess cyanobacterial endosymbionts called “spheroid bodies” in addition to plastids and mitochondria^2,6,7^. Unlike other diatoms, *Rhopalodia* and *Epithemia* species containing the spheroid bodies can grow in media with little or no nitrogen source^7^, and the nitrogen fixation capacities in *R. gibba* and *E. turgida* were experimentally confirmed^3,8^. Consequently, it is now widely accepted that the spheroid bodies (cyanobacterial endosymbionts) in rhopalodiacean diatoms operate nitrogen fixation and supply nitrogen compounds to the host diatoms^8^. Indeed, molecular phylogenies confirmed a close relationship between the spheroid bodies and nitrogen-fixing cyanobacteria belonging to the genus *Cyanothece* ^6,8^.

The spheroid bodies inside cells of rhopalodiacean diatoms are separated from the cytoplasm by an envelope, which is thought to consist of two menbranes based on previous studies^7–9^. Although the spheroid bodies still retain the structural characteristic derived from their cyanobacterial ancestor such as thylakoid membranes, the intracellular structure bears neither prominent pigmentation nor chlorophyll autofluorescence^10^. The spheroid bodies are obligate endosymbionts, as these structures have never been cultivated outside of the host cells^8^ and are passed to the daughter cells through binary cell division^10,11^. Furthermore, both host (diatom) and symbiont (cyanobacteria) phylogenies suggested that the endosymbiosis of a nitrogen-fixing cyanobacterium, which later gave rise to the spheroid body, was established in the common ancestor of species in genera *Rhopalodia* and *Epithemia*, and inherited throughout the host speciation^6^. By taking into account the information from fossil records, the acquisition of the spheroid body could be traced back to the middle Miocene epoch, approximately 12 Mya^6^.

We have reported the complete genome sequence of a spheroid body in one of the rhopalodiacean diatoms, *Epithemia turgida* ^3^. The complete spheroid body genome sequence revealed an apparent reductive nature of the genome with regard to both the size and gene repertoire. Strikingly, the spheroid body genome in *E. turgida* appeared to lack most of the genes involved in photosynthesis, indicating that an autotrophic lifestyle is impossible for the cyanobacterium-derived intracellular structure. In contrast, the genes related to nitrogen fixation were well conserved in the spheroid body genome, consistent with the proposed function of the intracellular structure in the diatom cell. The first complete spheroid body genome sequence illuminated recent metabolic adaptations to an intracellular lifestyle as well as genome reductions driven by metabolic adaptations. However, except the spheroid body genome of *E. turgida*, only a partial genomic data from the spheroid body of *Rhopalodia gibba* was available^12^. The knowledge learned from the single complete spheroid body genome may not be sufficient to depict the evolutionary process worked on the endosymbiont and its genome during transition from a cyanobacterial endosymbiont to a host-controlled intracellular structure. To explore the genetic and metabolic divergences among the spheroid bodies and the dynamics in the reductive process shaped the extant spheroid body genomes, here we report the complete genome sequence for the spheroid body of *Rhopalodia gibberula*, which is genetically and morphologically distinct from *E. turgida*. The detailed comparison between the *E. turgida* and *R. gibberula* spheroid body genomes provided new insights into the genome evolution of the obligate cyanobacterial endosymbiont in rhopalodiacean diatoms.

## Results and Discussion

### Overall difference between the two spheroid body genomes

**Figure 1:**
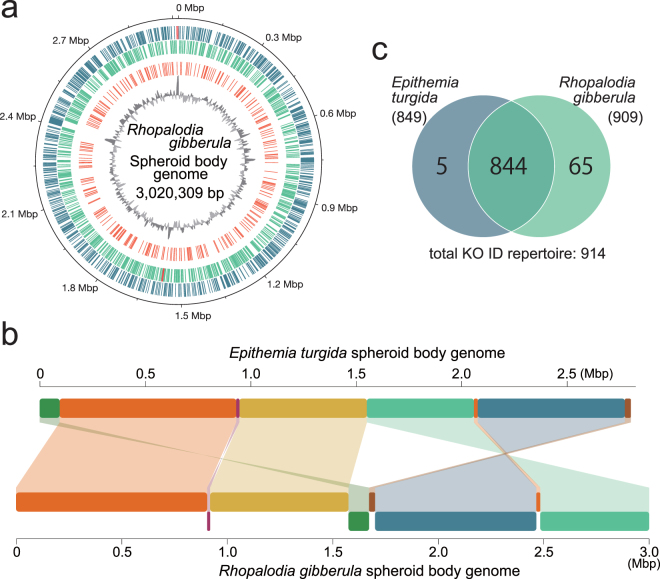
The spheroid body genome of *Rhopalodia gibberula* and comparison with the spheroid body genome of *Epithemia turgida*. (**a**) Map of the circular chromosome of the spheroid body of the diatom *Rhopalodia gibberula* (*Rg*SB). Dark and light green bars in the outermost and second outermost circles show the positions of protein-coding genes on the plus and minus strands, respectively. The two circles also contain rRNA gene clusters shown by red bars. The bars on the second innermost circle indicate pseudogenes, and the innermost circle shows the GC content (window size: 5,000 bp). (**b**) Whole-genome comparison between the *Rg*SB and *Epithemia turgida* spheroid body (*Et*SB). The *Rg*SB genome sequence was aligned based on the *Et*SB genome sequence. Syntenic regions between the two genomes are colour-coded. The regions coloured in purple, dark green, light green and dark blue were found to be inverted in the *Rg*SB genome relative to the *Et*SB genome. (**c)** Comparison of the unique KEGG orthology (KO) ID repertoire of the *Rg*SB and *Et*SB genomes. The numbers in parentheses indicate the total number of unique KO IDs in each genome.

A culture strain of *Rhopalodia gibberula* was established from a sample collected in a fresh water pond at Tsukuba city, Japan. DNA extracted from a spheroid body-enriched cellular fraction of *R. gibberula* was amplified and analysed with Illumina MiSeq. After assembling short reads from the sequencing and gap-filling by PCR, we successfully obtained a single circular chromosome for the spheroid body of *R. gibberula* (*Rg*SB). The complete *Rg*SB genome was found to be ~3.02 Mbp in size (Fig. 1a, Table 1), which is over 200 Kbp larger than the previously reported genome of the spheroid body of *E. turgida* (*Et*SB)^3^, but smaller than the genomes of free-living cyanobacteria closely related to the spheroid bodies, e.g., *Cyanothece* sp. PCC 8801 (4.68 Mbp^13^) and *Cyanothece* sp. ATCC 51142 (5.36 Mbp^14^). The number of rRNA gene clusters and tRNA genes were identical between the *Rg*SB and *Et*SB genomes (2 and 39, respectively; Table 1). The GC content was almost identical between the two spheroid body genomes (33–34%; Table 1). Although the size of the *Rg*SB genome is larger than that of the *Et*SB genome by >200 Kbp, the two genomes appeared to be similar to each other as a whole and no large sequence blocks unique to the *Rg*SB genome were found (Fig. 1b). The spheroid body genomes have been rearranged during the divergence of rhopalodiacean diatoms, as inversions and/or translocations were detected between the two spheroid body genomes (Fig. 1b). 1,671 and 1,720 open reading frames (ORFs) were predicted in the *Rg*SB and *Et*SB genomes (Tables S1 and S2), respectively, and 60.3 and 54.5% of the predicted ORFs (i.e. 1,007 and 937 ORFs) in the *Rg*SB and *Et*SB genomes, respectively, were assigned into any of functional categories in the Kyoto Encyclopedia of Genes and Genomes (KEGG) orthology (KO). We found that the majority of KO IDs from the *Rg*SB and *Et*SB genomes were shared between the two genomes (844 out of 909 and 849, respectively; Fig. 1c). However, the number of unique KO IDs was found to be different between the two genomes. The *Rg*SB genome had 65 KO IDs that were absent in the *Rg*SB genome, while only five KO IDs were unique to the *Et*SB genome (Fig. 1c). Increment of insertion sequence (IS) is believed to play a key role in the genome reduction^15^. We found 22 and 18 IS elements in the *Rg*SB and *Et*SB genomes, respectively. None of the IS elements found in the two spheroid body genomes are likely transposable, as their internal ORFs for transposases are fragmented or severely truncated.

**Table 1:** Genome overview of the spheroid bodies of *Rhopalodia gibberula* and *Epithemia turgida*, and two closely related free-living cyanobacteria.

	Spheroid body of *Rhopalodia gibberula*	Spheroid body of *Epithemia turgida*	*Cyanothece* sp. PCC 8801	*Cyanothece* sp. ATCC 51142
Genome size (bp)*	3,020,309	2,794,318	4,679,413	5,363,972
GC content	33.9%	33.4%	39.8%	37.9%
rRNA gene cluster	2	2	2	2
tRNA gene	39	39	43	43
Protein coding genes	1,671	1,720	4,367	5,304
Functionally annotated**	1,007 (60.3%)	937 (54.5%)	1,659 (38.0%)	1,727 (32.6%)
Protein with ambiguous function**	664 (39.8%)	783 (45.5%)	2,708 (62.0%)	3,577 (67.4%)
Pseudogenes	286	225	199	6
Reference	This study	Nakayama *et al*.^3^	Bandyopadhyay *et al*.^13^	Welsh *et al*.^14^

^*^Values for main chromosomes.

**Values in parentheses indicate percentages among the total protein-coding genes of each genome.

Besides the circular chromosome of the *Rg*SB, we also obtained a ~6 Kbp contig (Dataset S1) carrying ORFs identified in previously sequenced cyanobacterial genomes. The fragment was closely related to the ~5.7 Kbp contig found in the *Et*SB genome sequencing^3^, suggesting that the fragment is conserved between the two distinct rhopalodiacean species. These fragments may correspond to plasmids of the spheroid bodies, albeit we are still unsure of their cellular localization at this point.

### Pseudogenisation is less advanced in the *Rg*SB genome than the *Et*SB genome

The complete genome sequence of the *Et*SB demonstrated that the symbiont has lost its photosynthetic ability entirely as a consequence of adaptations to an endosymbiotic lifestyle^3^. Most genes for components of photosystems I and II as well as other proteins essential for photosynthesis appeared to be absent in the *Et*SB genome. Consistent with the non-photosynthetic nature of the *Et*SB, entire genes for the chlorophyll *a* (Chl-*a*) biosynthetic pathway were found to be pseudogenised or undetected in the genome. The Calvin cycle also appeared to be incomplete because both *rbcL* and *rbcS* encoding the large and small subunits, respectively, of ribulose-1,5-bisphosphate carboxylase/oxygenase (RuBisCO) were pseudogenised. The putative metabolic functions retained in the *Rg*SB, which were reconstructed from the genome sequence generated in this study, were essentially identical to those retained in the *Et*SB. For instance, we failed to find any functional genes for photosystem I or II in the *Rg*SB genome, indicating that the symbiont lacks photosynthetic ability. Nevertheless, we noticed that genes, which became dispensable for an endosymbiotic lifestyle, were degraded more rapidly in the *Et*SB genome than the *Rg*SB genome. The most prominent examples are the biosynthetic pathways for Chl-*a* and vitamin B_12_, which are described below in detail.

**Figure 2:**
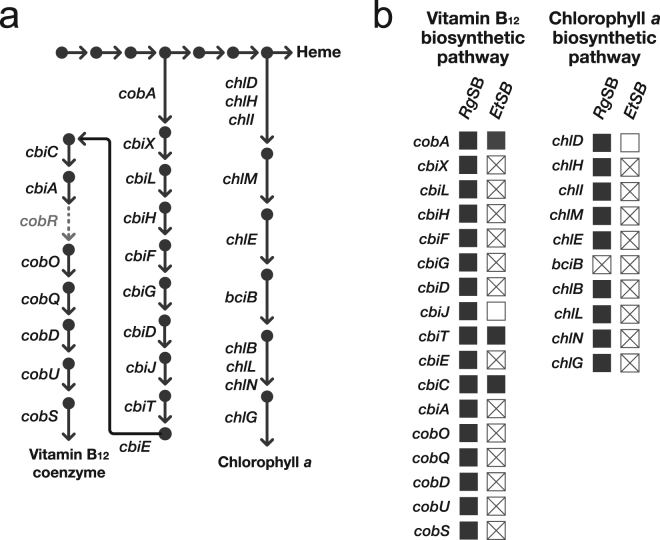
Chlorophyll *a* and vitamin B_12_ biosynthetic pathways in the spheroid bodies of *Rhopalodia gibberula* (*Rg*SB) and *Epithemia turgida* (*Et*SB). (**a**) Pathways for chlorophyll *a* and vitamin B_12_ biosynthesis. Each arrow indicates a single reaction. Gene names corresponding to each reaction are displayed. The step indicated by a dashed grey arrow is catalysed by cobyrinic acid *a*,*c*-diamide reductase (encoded by *cobR*) in diverse photosynthetic organisms, but this gene was not detected in the genome of the *Rg*SB, *Et*SB, or their close free-living relatives (i.e. *Cyanothece* spp.). (**b**) Status of genes for the chlorophyll *a* and vitamin B_12_ biosynthetic pathways in the *Rg*SB and *Et*SB genomes. A filled box indicates the presence of an intact gene, whereas a box with a X mark and a blank box designate the presence of a pseudogene and the absence of a gene, respectively.

Chl-*a* and vitamin B_12_ possess tetrapyrrole rings as their backbones, and these biosynthetic pathways branch from the heme biosynthetic pathway (Fig. 2a). We identified all of the 10 genes that composes the Chl-*a* biosynthetic pathway in the *Rg*SB genome. Consistent with the non-photosynthetic nature of the *Et*SB, all of these genes were predicted to be dysfunctional in the *Et*SB genome^3^. In contrast, 9 out of the 10 genes (*chlB*, *chlD*, *chlE, chlG*, *chlH*, *chlI*, *chlL*, *chlM*, and *chlN*) in the *Rg*SB genome appeared to bear no apparent signs of dysfunctionalisation (Fig. 2b, Table S3). A single pseudogene in the Chl-*a* biosynthetic pathway in the *Rg*SB genome encoded cyanobacterial 3,8-divinyl chlorophyllide reductase (*bciB*)^16^, of which the ORF was disrupted by a frame shift and in-frame stop codons (Fig. 2b). These data suggest that the *Rg*SB cannot produce Chl-*a* due to the lack of a functional *bciB*, but other genes involved in the corresponding pathway carry intact ORFs. Our analysis on the non-synonymous substitution rates (dN values) revealed dN values of genes for the Chl-*a* biosynthesis were found to be significantly greater than the values from other genes in the *Rg*SB genome (Figure S1). This observation suggests that the functional constraint on the Chl-*a* biosynthetic pathway as a whole has been loosened, implying that the genes involved in this pathway have already been pseudogenised albeit without apparent signs of dysfunctionalisation in their primary structure. In the case of the vitamin B_12_ biosynthetic pathway, 17 out of the 18 genes were identified in the *Rg*SB genome (Table S3). Note that *cobR*, which encodes cobyrinic acid *a*,*c*-diamide reductase, was excluded from the discussion below, as this gene was not identified in the two spheroid body genomes or their free-living relatives such as *Cyanothece* spp. The *Et*SB cannot synthesize vitamin B_12_, as 14 of the 17 genes were found to be pseudogenized or undetected in the genome (Fig. 2b, Table S3). On the other hand, none of the same set of genes appeared to bear any obvious signs of dysfunctionalisation in the *Rg*SB genome (Fig. 2b), implying that the *Rg*SB can synthesize the vitamin B_12_ coenzyme by itself. In support of this idea, the dN values calculated from genes for the vitamin B_12_ biosynthesis showed no significant elevation when compared to the values of genes for Chl-*a* biosynthesis (Figure S1).

Vitamin B_12_ synthesized in the *Rg*SB can be utilized for vitamin B_12_-dependent enzymes. We found *metH* encoding a vitamin B_12_-dependent enzyme, methionine synthase (5-methyltetrahydrofolate-homocysteine methyltransferase), which catalyzes the final reaction of the methionine biosynthesis, in the *Rg*SB genome (locus ID: RGRSB_0352). This enzyme is most likely functional in the *Rg*SB, as (i) its amino acid sequence bears a high identity to the orthologue in *Cyanothece* sp. PCC 8801, a free-living relative of the spheroid bodies (Figure S2), and (ii) the dN value for *metH* was not significantly high as it was not detected as an outlier (the Grubbs’ test, *P* < 0.005). Interestingly, we noticed the possibility of methionine synthase being also functional in the *Et*SB, which cannot synthesize vitamin B_12_ by itself. *metH* appeared to be retained intact in the *Et*SB genome (locus ID: ETSB_0394), and its amino acid sequence keeps high identities to both orthologues in the*Rg*SB and free-living cyanobacteria (Figure S2). If methionine synthase is genuinely functional in the *Et*SB, the intracellular structure need to uptake external vitamin B_12_. Diverse bacteria including *Cyanothece* spp. uptake vitamin B_12_ by BtuCD-F transport system, one of ABC transporters^17^. Nevertheless, no gene for the BtuCD-F transport system was found in the *Et*SB (or *Rg*SB) genome. Thus, the *Et*SB uptakes external vitamin B_12_ by either of the two systems described below—1) an as-yet-unidentified transporter system equipped originally with the spheroid bodies or 2) the transporter supplied from the host (diatom) cell. If the latter is the case, it is attractive to speculate that the genes for the BtuCD-F transport system were relocated from the endosymbiont genome to the host genome (i.e., endosymbiotic gene transfer or EGT^18^), and the gene products are now targeted to the envelope of the *Et*SB. Alternatively, the host supplies a transporter system of non-cyanobacterial origin to the *Et*SB to uptake vitamin B_12_. Future studies on vitamin B_12_ transport in *E. turgida* and its spheroid body may provide keys to judge whether EGT occurred in the rhopalodiacean diatom system. To tackle the above issue, both genome data of the host diatoms and proteomic data of the spheroid body envelope are indispensable.

### Difference in non-synonymous substitution rate between the two spheroid body genomes

**Figure 3:**
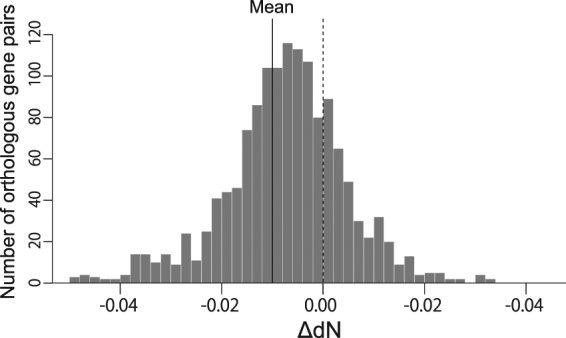
Overall difference in non-synonymous substitution rates between the *Rhopalodia gibberula* spheroid body (*Rg*SB) and *Epithemia turgida* spheroid body (*Et*SB) genomes. For each orthologous pair of the *Et*SB and *Rg*SB genes, non-synonymous substitution rates (dN values) were estimated, and then the difference between the two corresponding dN values (ΔdN) were calculated. To calculate ΔdN, the dN values of a *Et*SB gene was subtracted from the value of the corresponding gene in the *Rg*SB genome. The mean of the total ΔdN values was -0.0101 (indicated by a solid line). Outliers were not included in this histogram.

In the previous section, we focused on two metabolic pathways with differential tempo of pseudogenisation between the *Rg*SB and *Et*SB genomes. In this section, we explore the evolutionary mechanism underlying the difference observed between the two spheroid body genomes by calculating and comparing the evolutionary rates of the genes shared between the two genomes. 1,450 orthologous genes were extracted from the two spheroid body genomes, and the number of non-synonymous substitutions from the most recent common ancestor was estimated. The differences in dN values (ΔdN), which were calculated by subtracting the *Et*SB values from the corresponding *Rg*SB values, were found to be almost normally distributed, but the entire distribution was skewed toward the negative side (Fig. 3). We conducted the Wilcoxon signed-rank test to examine the null hypothesis, which assumes that the dN values were not significantly different between the paired *Rg*SB and *Et*SB genes (i.e., ΔdN = 0). The null hypothesis was rejected at the 0.1% level (*P* < 0.001), suggesting that the *Et*SB genes tended to have evolved at higher non-synonymous substitution rates than the *Rg*SB genes. The difference in dN value between the *Rg*SB and *Et*SB genomes explains well the difference in tempo of pseudogenisation between the two genomes (Fig. 2b). In the ancestral spheroid body genome, a large number of genes should have become dispensable along with the transition from a photosynthetic/free-living to a non-photosynthetic/endosymbiotic lifestyle. Nevertheless, the fates of the dispensable genes most likely varied between the descendent genomes evolving with different evolutionary rates. The chance to receive apparent signs of dysfunctionalisation (e.g., nonsense substitutions) was most likely higher in the *Et*SB genome than that of *Rg*SB genome, leading to the difference in the Chl-*a* and vitamin B_12_ biosynthetic pathways observed between the two genomes (see the previous section).

## Conclusion

Obligate bacterial endosymbionts in unicellular eukaryotes are regarded as models to retrace the evolutionary transition from a free-living bacterium to an organelle integrated into the host eukaryotic cell. In this regard, the spheroid bodies in rhopalodiacean diatoms are expected to provide unique evolutionary insights into how the intracellular structures specialized for nitrogen fixation emerged through endosymbiosis. The current study on the two spheroid body genomes demonstrated that a certain degree of diversity exists among the endosymbiont genomes in the diatoms. Such genomic diversity has also been reported from cyanobacterium-derived structures/endosymbionts recently integrated into eukaryotic cells: (i) cyanobacterium-derived structures (chromatophores) in testate amoebae *Paulinella* spp.^19–21^, and (ii) nitrogen-fixing obligate cyanobacterial endosymbionts (UCYN-A cyanobacteria) associated with a particular group of haptophytes^22,23^. We need to further assess the genomic diversity observed in the aforementioned endosymbionts carefully to separate the genomic changes, which were critical for obligate endosymbiotic lifestyles and organellogenesis, from random changes that accumulated in the endosymbiont genomes.

## Materials and Methods

### DNA preparation from the spheroid bodies of Rhopalodia gibberula cells

*Rhopalodia gibberula* cells were found in a sample collected from a pond in Namiki park, Tsukuba, Ibaraki, Japan (36°03.56 North, 140°08.32 East). A single cell was isolated by micropipetting and cultured clonally in nitrogen-depleted medium (CSi-N^6^). The cultured diatom cells were mildly disrupted by vortexing with glass beads (~1 mm diameter) for five minutes, and then the spheroid bodies were separated roughly from other intracellular particles by discontinuous density Percoll gradient centrifugation as previously described in Nakayama *et al*.^3^. After the centrifugation, the absence of the host DNA-containing organelles (i.e., diatom nuclei, mitochondria, and plastids) in the spheroid body-enriched fraction was confirmed under a light microscope. The spheroid body-enriched fraction was subjected to whole-genome amplification using the REPLI-g mini kit (Qiagen). The whole-genome amplicon was de-branched with S1 nuclease to reduce chimeric sequences during the amplification reaction.

### Genome sequencing and assembly

Amplified DNA was subjected to a library construction using a TruSeq Nano DNA sample preparation kit (Illumina) with 550 bp inserts, as manufacturer’s recommendations. The library analyzed on an Illumina MiSeq platform (300 bp, paired-end), yielding ~51 million short reads. The first 5 and last 50 bases of each read as well as reads with low sequencing quality were removed using FASTQ_Trimmer and FASTQ_Quality_Filter, respectively, which are both included in the FASTX_Toolkit program package (ver. 0.0.14; http://hannonlab.cshl.edu/fastx_toolkit/). Eight million paired-end reads were used for assembling the genome using SPAdes ver. 3.1.0^24^. Genome scaffolding with assembled contigs was performed with SSPACE^25^. To obtain candidate spheroid body genome sequences from the final scaffold pool, we performed BLASTn search using the *Et*SB genome^3^ as a query. Eleven large scaffolds with high sequence similarity to the *Et*SB genome were retrieved, and gaps between those scaffolds were closed by PCR with information from pairs of paired-end reads.

### Genome annotation

Predictions of the ORFs on the completed circular genome sequence were performed by using GeneMarkS^26^. The gene models were carefully inspected and refined manually. Transfer RNA genes were detected by tRNAscan-SE^27^, and rRNA genes were predicted based on nucleotide similarity. Putative pseudogenes on the spheroid body genome were identified by tBLASTn against non-coding regions of the genome using cyanobacterial proteins in the NCBI RefSeq database as queries. Coding regions interrupted by stop codons and/or disrupted by frame shifts as well as severely truncated ORFs were tagged as putative pseudogenes. IS elements in the genomes of the *Rg*SB and *Et*SB were initially identified by ISsaga, an IS identification system for prokaryotic genomes^28^. The output from ISsaga was manually checked and refined. A list for the detected IS-related sequences in our analysis is shown in Table S4.

The circular genome map was generated by using DNAplotter^29^. Structural comparisons between the *Et*SB and *Rg*SB genomes were performed with Mauve^30^. The initial KO ID assignment was performed by using the KEGG Automatic Annotation Server^31^ and then manually refined. The annotated genome data is available in DNA databases in Japan/GenBank/European Molecular Biology Laboratory under BioProject accession number PRJDB4388.

### Gene evolutionary rate

We estimated dN values of protein-coding genes in the *Rg*SB genome. Nucleotide sequences for 1,563 orthologous genes were extracted from genomes of the *Rg*SB, *Cyanothece* spp. PCC 8801, PCC 8802, and ATCC 51142 and then were aligned based on codons using an in-house ruby script and MAFFT^32^ with the L-INS-i option. The dN values were estimated with the CODEML implemented in PAML^33^ (settings: runmode = 0, seqtype = 1, CodonFreq = 2, model = 1) based on the organismal phylogenetic relationship among the *Rg*SB and its free-living relatives (i.e. *Cyanothece* spp; see Figure S3A). The dN values of the *Rg*SB genes, which are values for a branch from the node representing the most recent common ancestor of *Cyanothece* spp. PCC 8801, PCC 8802, and the *Rg*SB (branch X in Figure S3A), were standardized by the corresponding dN values for branch of *Cyanothece* spp., that is, the values from the node shared by the *Rg*SB to a node of the most recent common ancestor of two *Cyanothece* spp. (branch Y in Figure S3A; Table S5). The dN values that were impossible to standardize (i.e., dN values for the *Rg*SB or *Cyanothece* spp. gene = 0) were omitted from downstream analyses. Outliers among the dN values for the *Rg*SB genes were detected using the Grubbs’ test. Differences in the normalized dN values between the gene sets of the two biosynthetic pathways for Chl-*a* and vitamin B_12_, and total genes in the genome was tested by the Wilcoxon rank-sum test. In addition, we estimated non-synonymous substitution rates of protein-coding genes shared between the *Et*SB and *Rg*SB genomes. Nucleotide sequences for 1,450 orthologous genes were extracted from the two spheroid body genomes, *Cyanothece* spp. PCC 8801, PCC 8802, and ATCC 51142, and then analysed as described above (the tree topology used for the estimation is presented in Figure S3B). The dN values of the *Rg*SB and *Et*SB sequences for branches from the node representing the latest common ancestor (branches X and Y in Figure S3B) were compared (Table S6). To test the bias of the difference of dN values between *Rg*SB and *Et*SB genes, a Wilcoxon signed-rank test was performed.

## Electronic supplementary material

Supplementary Figures and Legends

Table S1

Table S2

Table S3

Table S4

Table S5

Table S6

Dataset S1
