# Supplementary material for: Genomic divergence within non-photosynthetic cyanobacterial endosymbionts in rhopalodiacean diatoms
Source: Sci Rep. 2017 Oct 12;7:13075. doi: 10.1038/s41598-017-13578-8 (PMC5638926; doi:10.1038/s41598-017-13578-8)
Supplement: Supplementary file 1 — Supplementary Figures and Legends [file 41598_2017_13578_MOESM1_ESM.pdf]

Scientific Reports—Article

Title:

Genomic divergence within non-photosynthetic cyanobacterial endosymbionts in rhopalodiacean diatoms

Authors: Takuro Nakayama* and Yuji Inagaki

Center for Computational Sciences, University of Tsukuba, 1-1-1 Tennoudai, Tsukuba, Ibaraki 305-8577, Japan

Supplementary Figure Legends

Figure S1. Normalized rates of non-synonymous substitution for total gene set and genes for the chlorophyll *a* and vitamin B_{12} biosynthetic pathways. Each boxplot represents the distribution of non-synonymous substitution rates (dN values) on protein-coding genes in the *Rg*SB genome, which were normalized by that of counterpart genes from *Cyanothece* spp. genomes (see material and method section in the main text). The difference in the normalized dN values between the total gene set and the Chl-*a* biosynthetic genes appeared to be significant (the Wilcoxon rank-sum test, $P = 0.00029$). On the other hand, no significant difference in the dN value distribution was observed in the same test considering the genes for vitamin B_{12} biosynthesis (the Wilcoxon rank-sum test, $P = 0.3277$). Outliers were not displayed in this graph.

Figure S2. Sequence comparison of the methionine synthase from the *Rg*SB, *Et*SB and free-living cyanobacteria. (a) Multiple-alignment of the amino acid sequences. (b) Pairwise identity matrix of sequences displayed in (a). The values indicate percentages of amino acid identity.

Figure S3. Phylogenetic tree topologies used in CODEML analyses.

Figure S1

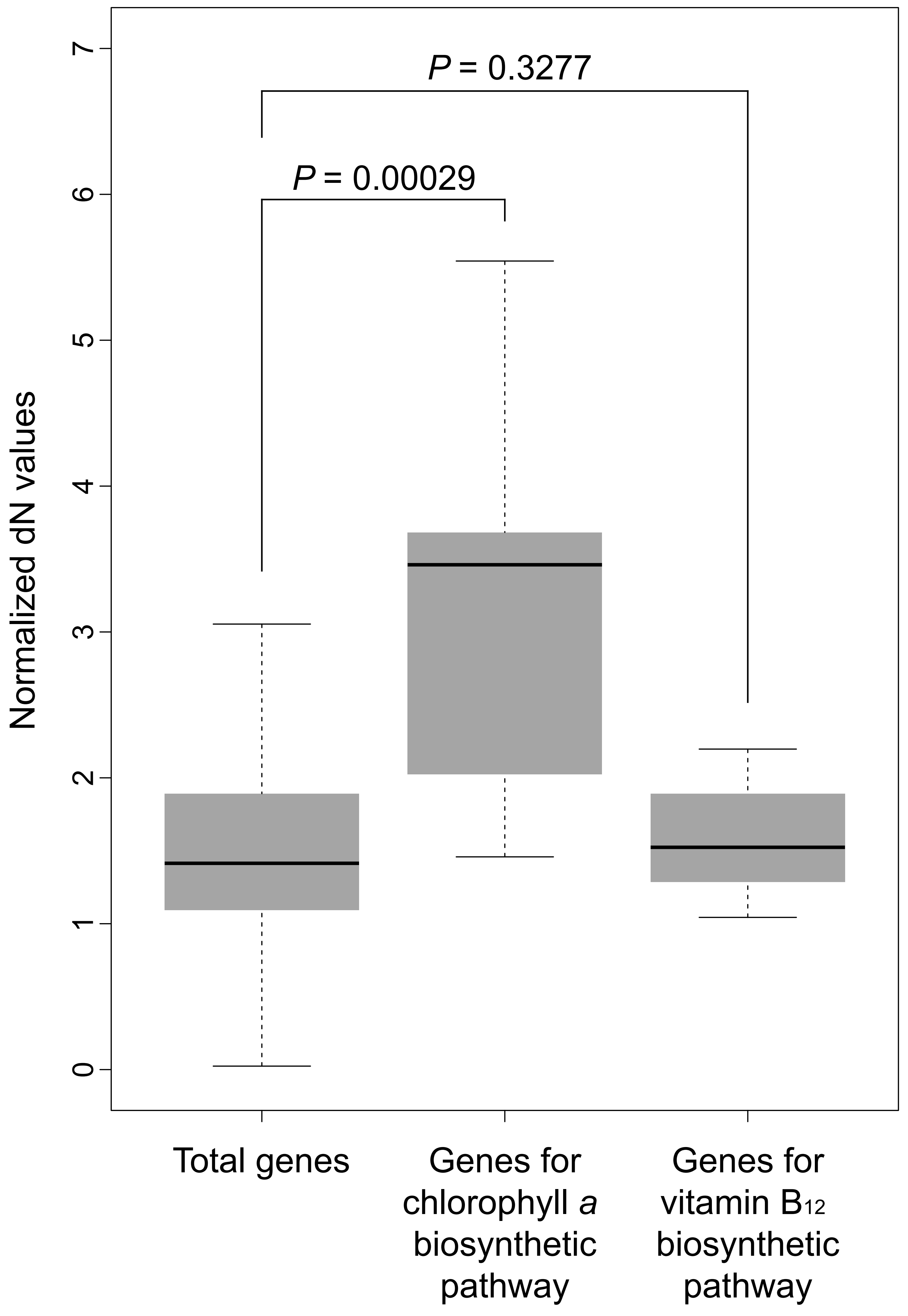

P = 0.3277
P = 0.00029
Normalized dN values
0
1
2
3
4
5
6
7
Total genes
Genes for chlorophyll a biosynthetic pathway
Genes for vitamin B12 biosynthetic pathway

Figure S2

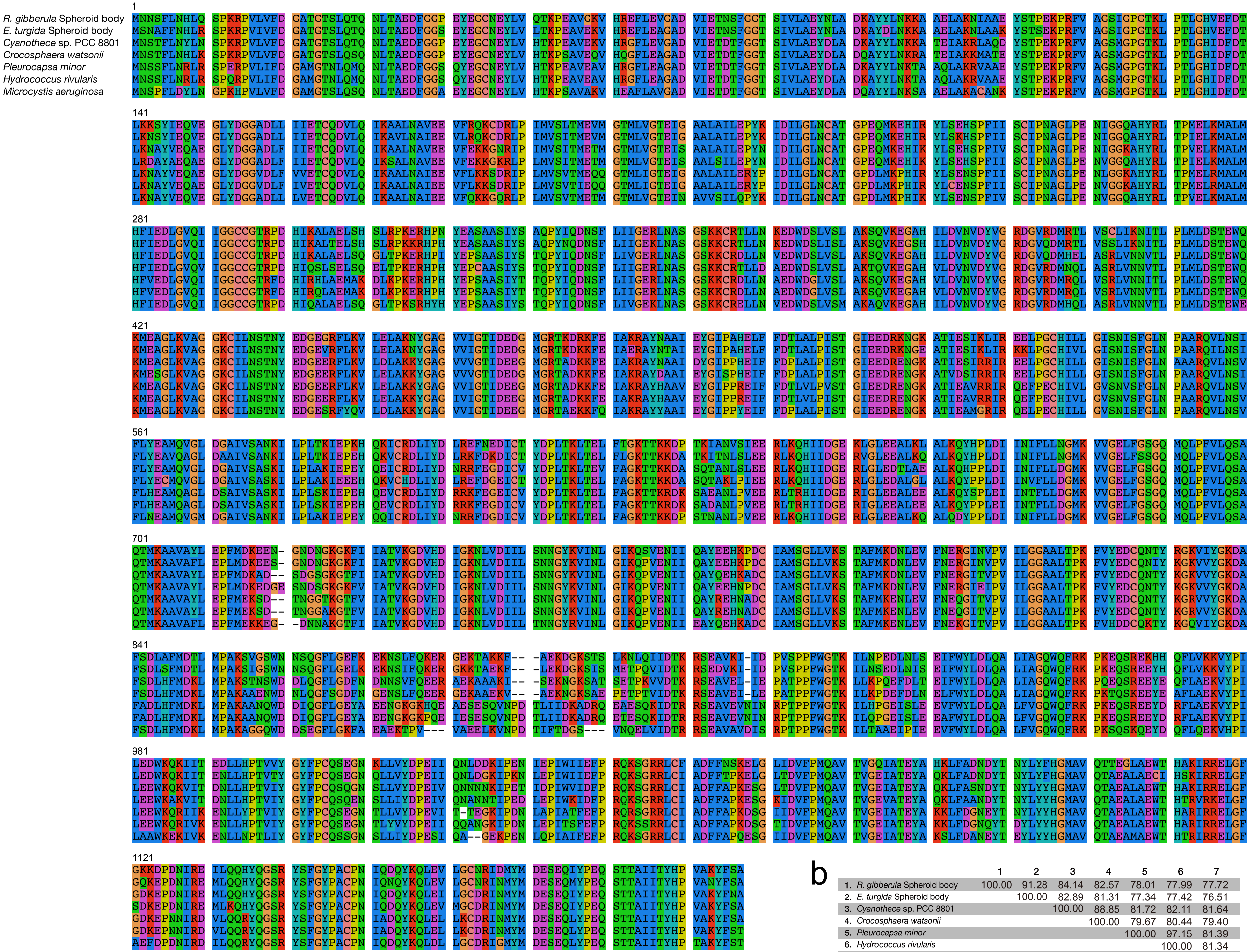

b

		1	2	3	4	5	6	7
1.	*R. gibberula* Spheroid body	100.00	91.28	84.14	82.57	78.01	77.99	77.72
2.	*E. turgida* Spheroid body		100.00	82.89	81.31	77.34	77.42	76.51
3.	*Cyanothece* sp. PCC 8801			100.00	88.85	81.72	82.11	81.64
4.	*Crocosphaera watsonii*				100.00	79.67	80.44	79.40
5.	*Pleurocapsa minor*					100.00	97.15	81.39
6.	*Hydrococcus rivularis*						100.00	81.34
7.	*Microcystis aeruginosa*							100.00

Figure S3

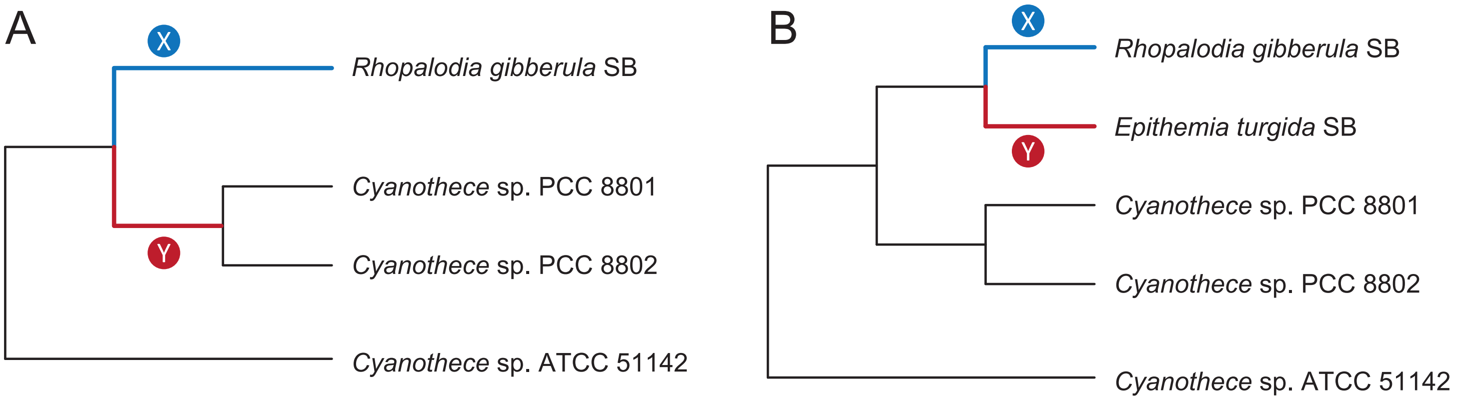

A
X
Y
Rhopalodia gibberula SB
Cyanothece sp. PCC 8801
Cyanothece sp. PCC 8802
Cyanothece sp. ATCC 51142
B
X
Y
Rhopalodia gibberula SB
Epithemia turgida SB
Cyanothece sp. PCC 8801
Cyanothece sp. PCC 8802
Cyanothece sp. ATCC 51142
