# Supplementary material for: Genomic divergence within non-photosynthetic cyanobacterial endosymbionts in rhopalodiacean diatoms
Source: Sci Rep. 2017 Oct 12;7:13075. doi: 10.1038/s41598-017-13578-8 (PMC5638926; doi:10.1038/s41598-017-13578-8)
Supplement: Supplementary file 8 — Dataset S1 [file 41598_2017_13578_MOESM8_ESM.doc]

>RgSB_P1

AGAAATTTGTAATTTATCTTAAATCTTAAGTATAAGTTATGATTTAACCGTTAAAATTAATTAAAAATTAATTACTAAAG

TTGAAGTCAATGTCAAAACATTCGAAACTCTTGTCAGAAGTTTTAGCTGAAATAATAGGTACTTTTATCTTATTATTTAC

TGGAATAGGAGCGATTATGGTCAATACAATTACAGAGGGTTTAATTACTCATCTCGGAATTTGTTTTATATTTGGTGCAG

TAGTTACAGCTATAATTTATACCATAGGACATATTAGTGGAGCTCATATTAATCCAGCAGTTACCCTAGCTTTTTGGAGT

AGTAAAGTTTTACCTAAAAATAAAGTATTGCCTTATATTATAGGTCAATTTAGTGGTGGAATTTTAGCTTTATTATTATT

GCGAATAATTTTTGGTAATATTGCTGATATGGGAGTTACTTTACCATTAGAAGGTAATTGGTTACAATCTTTAATTATAG

AAGTAATTTTAACTTTTATTTTGATGTTTGTTGTTTTAGGTTCAGGATTAGATCGTAGAGCTCACATTGGTTTTGCTGGA

ATAGCTGTTGGCTTAACAGTTAGTTTAGAGGCAGCTTTTATGGGTCCAATAAGCGGGGCTAGTATGAACCCAGTAAGATC

TTTAGCACCAGCATTAATTACTCACACATGGCAATATCAATGGTTATATATTATCGGTCCAATTGTTGGAGCTCAACTAG

CTGTCTGGATATACCGTCAATTATCAAATAATTTTCACGATTTTGACTCTAAAGAATTAGTTTAATTTTTTTAAATTTGT

TGTTAAAAAAAGTTAAAATTATAAAATATATAGATTAATTTTGTAATTTTAACTTTTTAATTTAAGTAAAATACTAACTA

AATTTAAAAAGTTATTAAACTATAAATTATAGTAATAATATTTAAGTTTCATCTTGATCAAGTTTATTTTCTTTAGATAA

AAAATCATTAGAAATGATATCAGTTGGATTGACTTGTTGTTTACTCTTTTTCTTTTTTAATTGATTTTTTTTATCAAAAC

TTAGTATCTCAAAGAGTGGTTTCTTATTTTTAAGTTTTTTAGTTTGAGGGATAGGATTTTCTTGATCTAAAGATAAAGAT

AAATTTATGTATTCCAAACTAGAGCTCTCTAAAATTTTTACTAAGTTAGGAGTTTCTATCGAAATCTGCTTTGTTTCATC

GCATAGATCTTGTTCTTCATTTTTTTCAGAAAGTGTGCCAATTGGCACACTTTCTGAATTTATCTGAACATGTTCTATTT

CATGGTTGGCAAAAGTTTCTAGATCCTCTTCCCATCTTGCTAAAGTTTCTTCCTTAACAATATCTACTGTTTGATCATCT

TCAAACAGCGAATTCAACAGTTGCTCAATAGAAAGCCCCATCTCAAGAGCTTTTCTTCTTATTTGCTCATATAGTTTTTT

AGAAAGTCGCAAACTAGCTGTCTTAGTTTCCACACCTAGTACCTCATTAATTTGATTACTTGTAATTGTATGCGGTTCAA

GGCTATCAACAACTGTTTTCCATTTATTACATAGTTCAGCTCCATATAGTTTAGTAAGAGGACGAGCTTGAGCCTCACAA

GTGGGAAGGACAGAAAATCCTGCTTGAGCTAATTCTATTACTACACGAGCAGCCTCAATTTTTCTATTAATTTGCCAATG

ACTAATTCCTAAAGCTTTTTCACAAAATGTTTTAAAATTCTTATGGACTCTACGATAAAGTTTTAAAATTTTTACTTTAT

AAGCTAACAAACCTGTGCGGACAAAAGACAATATTTGACTTTTAATCTCTGTCACAATCGTTTGTAAGTCAAAATCTCCA

GGTATACATTCTTCTAAGGACCAAGCGTAATGTATTAAATTTTCTATTGATTCACTGCGTAATATTTCTTCATCAGAGGT

TTGATAAAGAGAAGTGAAAGGATCTGCTAAAAAACTTTTTTTAGTTTTATAAGGATTTTGAATGCTACTAATGATAGTGT

CCATTGTCTTTCTACAAAATTAATAACTAAAGTTAGTAGAGACAAATAACACTGTTATGTGTTACCCTAAAATTGTTTAG

GCTTGAAGGGGGCACACGACATAAGTGTGTCTCTAACTATTTCTGGGCTATTATACGTTATAACGGTCTTAGTTTATAGT

TAAACAATTAGGTTAAATTTTTCAACAGTATTAAAAAATACTAAAAAGATTTTATAAAAAATTAAAGTTGAGTTAAGATT

AGTTCTTTTTGTGGAGTTTTAAAAATTCGAGATGGTATCGAAAAATTTTATTTTCGATACCATCTCGAATTTTTAGATAA

AAAATATTAAATTAATTTGGATATAATTCAGATATATCTAAATTAATTTAATATTTTTGAAAAGGTTAAAAGTTGTGTTC

CCTGATAAAAAATAAAACTAGTAATCCAAGCTGTTATAAGAGAAAACAATACTGACATAATGGTAAATTTAACAGAACGA

GATTCACCCCAGATAGTGCTAAGAGTAGTTAAACAAGGAATATATAATAAACTAAATAAACAATAGCTAAATCCCTGAGC

AAAAGTAAGATCCTGTCCAAGTTTGATGCCTAAAGCATCATTATTTAATCCATAAATGACTGCTAATCCTGCGAGTTGTA

CTTCTTTAGCAACAAAACCAAAAATTAAAGATACTGTCAAAAAAGGATTAATACCAATAGGATTCATAATAGGAGAGAAA

AATTTTCCTATTTGACCGGCCCAAGTATCTAGTCCTTCAGCTCCTAGTGGCAAAGTTGTCAATAACCAAATCAAACTTGT

TCCGATAAGCATAAATAACATTACTTTTGTAATAAATTGTTTTATTTCTCCCCAAACTTTTGTCACAATTTGTCTGAAAG

TAGGTAATCGGTAAGGAGGTAATTCTAAAACAAAGGGATCTTGACTTTTAAATTGATTACTAAGACTTAAAACTGCAGCA

ACGGCAAAAGCTACAAAAAAGCTAATCACATAAAGAGCAAATAAAGCAAAAGCACCGTTTTGATTAGGAAAAATAGCTGC

CAGAATAAATACAAATACTTGAAGACGTGCTGAACAAAGAGCTAAAGGAATAATTAGCATAGAAAGTAAACGCATTGTGC

GTGACCGCATGACTCTTGTTCCCATAATGGCTGGTACATTACAGCCAAATCCCATCATTTGTAATACAAAAGCTCGACCA

TCAAGTCCTAAATGCTTCATCAAAGCATCCATTAAATAAGCCGATCTAGAAAGATAACCACTATCTTCCAATGTTGACAT

AATTACAAAAAATAGAGCAACTAATGGTAAAAAAGATAAAATTGTAGCAAATCCATTCCATAAACCATTAATTATAAAAT

CTTGAATAATTTTAGGGAAAAAAGCAATAATTAAGATTAAAATATGTTCTTGAAACCAATTAGTTATAGTATCTATTGGA

CCAGCAGTAGGTATTCCTACTGACCAAAGAAACCAAAATACCATAAACATAATTGAGAAAAATAAAGGAAGTCCAAAAAA

TGGATGTAGTAACCATTTATCCAAACGATTAGTAAAAGTCATTTGGGTTAAAGGTGGCATTTGAACAGTTTTTTGTAATA

TCGACTCTATTTTTGATTCAGTTATTGGATTTGTTTTAAGATATTCAACAAGATGGTTAATTTTATGTGCATGAGATTGT

TTATTTAAAGCACGAATAATACCGCTAAGAGCTATATCACATCCAAAACCATATTTAGCACTTACAGAAAAAATAGGCAT

ACCCAAAAATTCACTAAGTTTAACTTTATTTATAAGTACTCCATAGCTTTTTGCTTCATCTACCATATTAAGAATTACCA

CTGCAGGAAATCCTAAAAGTTTTATTTGTAGTGCGATTCTAATTTGTCGATCAATTTGAGAAGCATTAATGACTACTACA

ACTAAATCTATTGGATAATTTTCTAAAAACTCTTGTACTACTTTTTCATCTTCTGTAAATCCGTCAAGATCGTAAATTCC

AGGTAAATCGACAAATTCAACTAGTTGGCCATCTAAATTAATTTCTGCTTGTAATAATTCAACTGTCAATCCAGGCCAGT

TAGCTACACTCGCTCCAGCATTAGTAATTCGATTAAAGAAAGTTGATTTCCCTGTATTAGGCTGACCTATAAAACTAATG

CGTTTTTTACTATTTTTTTGAGGAAAAACAGTAGTTTTCGGTTTTTTAGATATCGTCATATTTTTATATTTTTTTATTAG

TCATTATAGTGGTTCGATAATTACTAATTCTGCTTCACTTCGTCGAATAACAATCTCTGTTGTACTACCTACTCGTACCA

CTAATGGTCCTCCTAACCATGCAGCATGTATAACTTGAACTGGTTTTCCAGGTACTAATCCCATAGCTTCAAGTCGAATT

TTAAAACCTTTTCCTATCGAAGTACCATCATCTATGACTTTTTGAACAGTAGCAATACTTCCAATTTCTAATTCTCTTAA

AGTCATAATTTTAAATTATAATTTTTTTACTTTAAATAAAACTATGAATTATTTTTTATTTTAAAAATAACTATTATATT

TTTAATTAAATTAAAAAGTTAAGTTAAACTTTATTAAATAAATCTACAAACATGTTAAACTCTTATTTATTAGTATAGAT

TATAAAAAATTAATTTTTCAACTAATTTTTTATAATCTATTATCTAACATTTTAGACAATATAATTTAACTTATTAAGAT

TATAATTTTCCTATTAAATAATAAAAAAATTATATCAATAATATAGATAGTGAATTTCCTTAATAAAAGATTAACAGTAA

CTAATTTTTTTGTCAATTAATTTTTAAACTTATCAAAATATTTAAAAAATAAGATATATGATTATTTTATACTTATTAAT

ACCTATATATTTTAAATAAAATATATAGGTATTAATAAGTATAAAATTTAAAATTTTTTCTATCATTATTAGTTATAATA

ATGATATTTACTTCCGTATAAAAAAATCATGAATATATCTATTAAGTCCAATACTAATTCACCTTTAACTCACCAAGTTA

TTTCTCTTTGGCTGGCAGATAAACCTTCAGAAAAAACTCGTTATGAATATAAAAAAGATTTATATTATTTTTTTCAAAAA

ATATCTAATAGTGAAATTTCTGAAGATTTAATTAATAGTTTTTTAAAAATTACTCAGGCGCAAGCGAATATAATTTTAAT

AGCTTATAAAGAACACTTACGAAAACTAAAATTAGCTCCTACTACTATTAATAGAAAAATTAGTAGTATAAAATCCTTTA

TTTCAATAGCAAATCGTTTAAATTTATGTCATTTTTCTTTAAAAAATACAGTTTCTTCAGAAAGATTTAAATCTTATCGA

GATACATCAGGAATTTCTCTTTTAGAATTTAAAAAAGTTTTAACTTTATGTAAAACCTCTTCTTTAAAAGGAAAACGAGA

TTTTGCATTATTATTACTACTATGGACAAATGCTTTACGACGTAATGAAATTAGTTTATTAAATATAGAGGATTTTAAAC

CATCAAGGAATAAATTATGGATAAAAGGAAAAGGATATAATGAGAAACAAATTATAGATTTATCACCAAAAACAATTGAA

GCTATAGTTGATTGGTTAAAAAGTCGTAAAAATCAAAAAATTAACTATTATTCACCTTTATTTACTGCTTTAGATACAAA

ATCTTTTGGAAAAAGATTAACAGGTGACGGGATTTATAAAATTGTTCATCAATATTGTAAAAAAGCAGGAATTGATAAAC

AAATGTCTCCTCATCGTATTCGCCATTCGTCAATTACAGTAGCTTTAGATAAAAGTCAAGGAAATATTAGAAAAGTTCAA

AAATTATCAAGACATAAAAATTTAAATACTTTGATAGTTTATGATGATAATCGTAGACAAGATCAATTAGAGTTAAGTAA

ACAGCTTGAAGAAGATTTATTTTTAAATAATTAAAATTCATTTAATAAAAAATTAAATTCTGTTAAGGGATAATTAATTA

AGAAATTTGTAATTTATCTTAAATCTTAAGTATAAGTTATGATTTAACCGTTAAAATTAATTAAAAATTAATTACTA
